# Antimicrobial and Antioxidant Activities of *Pycnocycla spinosa* Extracts

**DOI:** 10.17795/jjnpp-13859

**Published:** 2014-06-22

**Authors:** Mohaddese Mahboubi, Mohsen Taghizadeh, Nastaran Kazempour

**Affiliations:** 1 Department of Microbiology, Medicinal Plant Research Center of Barij, Kashan, IR Iran; 2 Biochemistry and Nutrition in Metabolic Disorders Research Center, Kashan University of Medical Sciences, Kashan, IR Iran

**Keywords:** *Pycnocycla spinosa*, Extract, Antimicrobial Activity, Antioxidant Activity

## Abstract

**Background:**

*Pycnocycla spinosa (P. spinosa)* a member of the Umbelliferae family is traditionally used for treatment of different ailments.

**Objectives:**

This study aimed to evaluate the total phenolic and flavonoid content of *P. spinosa* extracts (methanol, ethanol and aqueous) and their antioxidant and antimicrobial effects.

**Materials and Methods:**

The antimicrobial activity of different extracts of *P. spinosa* was evaluated using micro broth dilution. Total phenolic and flavonoid contents were measured. Their antioxidant effect was evaluated using DPPH assay and β-carotene linoleic acid test.

**Results:**

*P. spinosa* ethanol extract with higher-level phenolic and flavonoid contents showed the highest antioxidant and antimicrobial effects, in comparison with the other extracts. *Bacillus* sp. and *Streptococcus* sp. showed higher sensitivity to *P. spinosa* ethanol extract.

**Conclusions:**

*P. spinosa* ethanol extract can be used as a mouthwash for treatment of the oral infections. More clinical and toxicological studies are required for providing its efficacy.

## 1. Background

Today, antibiotic-resistant microorganisms and side effects of these antibiotics concern the scientists. Therefore, they have been encouraged to find new sources of antimicrobial agents, especially among the medicinal plants.

Among different medicinal plants, *Pycnocycla spinosa* was the subject of a few investigations. *Pycnocycla* sp. belongs to the Umbelliferae family which has eight endemic perennial species with spine leaves in Iran (1). *P. spinosa,* as a member of this family is traditionally used for treatment of injuries and piles. Some pharmacological properties of *P. spinosa* aerial and root parts, like anti-spasmolytic and anti-diarrhea effects (2) and relaxant (3) and cardiovascular activity (4) have been confirmed. There are some reports on chemical composition of *P. spinosa* essential oil (5, 6). *P. spinosa* aerial part essential oil, contain caryophyllene oxide, β-eudesmol and α-cadinol (5). Elemicin (65%), linalyl acetate (11%), β-caryophyllene (7%) and β-eudesmol (4%) were the main constituents of *P. spinosa* seed essential oil (6).

## 2. Objectives

This is the first report on antimicrobial effects of different extracts (methanol, ethanol and aqueous extracts) from *P. spinosa* aerial parts, against microorganisms, including Gram-positive and Gram-negative bacteria, yeast and fungi in vitro condition. This study also evaluated the total phenolic and flavonoid contents of extracts and their antioxidant effects.

## 3. Materials and Methods

### 3.1. Plant Materials, Extraction, Total Phenolic and Total Flavonoid Contents

*P. spinosa* dried aerial parts were collected from Qamsar Road, Kashan, Iran, at a full flowering stage in March 2012 and authenticated under number 163-1. The powdered dried aerial parts of *P. spinosa* were mixed with each solvent (water, methanol and ethanol-water [70:30 v/v]) for 24 hours, at the ambient temperature, in the percolator (Taghtiran Company, Kashan, Iran). The mixture was separated and the extract was dried under a vacuum. Total phenolic content (TPC) and total flavonoid content (TFC) of each extract were determined using a procedure described later (7). TPC and TFC were reported as milligram gallic acid (GAC) and quercetin (QE) per gram of the dry extract, respectively.

### 3.2. Antioxidant Activity Evaluation

Radical scavenging effects of the extracts were determined based on reducing the 1,1-diphenyl-2-picrylhydrazyl (DPPH) (Sigma) solution. Different concentrations of the extract were added to 2 mL of a methanol (Merck) solution of DPPH. The absorbance of the solution was read against a blank at 517 nm (Spectrophotometer Jenuway, England), after a 70 minute incubation period, at room temperature. Inhibition of free radicals by DPPH was calculated in percent (I%) as follows: I% = [A_blank_-A_sample_/A_blank_] × 100.

Where A_blank_ is the absorbance of the control reaction (containing all reagents, except the test compound) and A_sample_ is the absorbance of the test compound. BHT (Sigma) was used as a positive control. The IC_50_ was determined as the concentration of a compound that inhibits 50% of the DPPH solution. Tests were performed in triplicate (7).

### 3.3. Carotene/Linoleic Acid Bleaching Test (BCB)

The β-carotene had a bleaching activity on the extracts (8). β-carotene (Sigma) was dissolved in chloroform (Merck) and then the chloroform was removed under vacuum, at 40°C. Afterwards the linoleic acid (Merck), Tween 80 (Merck) and aerated distilled water were added to the flask with vigorous shaking. The emulsion was added into a tube containing the extract and the absorbance, immediately and after 120 minutes at 470 nm, (after oxidation in a water bath at 50°C) were measured. The inhibition percentages (I%) of the samples were determined, using the following equation:

I% = (A_β-carotene_ after 2 hours assay/A_initial β-carotene_) × 100

A_β-carotene_ is the absorbance of β-carotene and A_initial β-carotene_ is the absorbance of β-carotene at the beginning of the experiments.

### 3.4. Microbial Strains and Antimicrobial Evaluation

The antimicrobial effects of the extracts were evaluated against many microorganisms from American Type Culture Collection (ATCC), including Gram-positive bacteria: *Staphylococcus aureus* ATCC 25923, *Staphylococcus epidermidis* ATCC 14490, *Staphylococcus saprophyticus* ATCC 15305, *Streptococcus pyogenes* ATCC 8668, *Streptococcus mutans* ATCC 35668, *Streptococcus sobrinus* ATCC 27607, *Streptococcus sanguis* ATCC 10556, *Streptococcus salivarius* ATCC 9222, *Streptococcus pneumoniae* ATCC 33400, clinical isolate of *Streptococcus agalactiae*, *Enterococcus faecium* ATCC 25778, *Enterococcus faecalis* ATCC 29212, *Bacillus cereus* ATCC 1247,* Bacillus subtilis* ATCC 6051, Gram-negative bacteria: *Klebsiella pneumoniae* ATCC 10031, *Escherichia coli* ATCC 8739, *Salmonella typhimurium* ATCC 14028, *Shigella dysenteriae* PTCC 1188, *Shigella flexeneri* PTCC 1234, *Pseudomonas aeruginosa* ATCC 9027, *Enterobacter aerogenes* NCTC 10006, yeast: *Candida albicans* ATCC 10231, *Candida glabrata* ATCC 90030 and fungi: *Aspergillus flavus*, *Aspergillus niger* ATCC 16404 and *Aspergillus parasiticus* ATCC 15517. The microbial suspensions were prepared in normal saline and their turbidity was adjusted to 0.5 McFarland. Afterwards, the microbial suspensions were diluted for further examination. The antimicrobial evaluation was performed, assessing the minimal inhibitory concentration (MIC) and minimal lethal concentration (MLC). The dried extracts were dissolved in each solvent, then the dilutions were prepared at the ranges of 51.2-0.1 mg/mL in sterile water and then 100 µL of each dilution was added into each well of 96 micro titer plates. This concentration is the best concentration for antimicrobial evaluating. One hundred microliter of diluted microbial suspension was added to each well and then incubated at 37ºC for 24 and 48 hours (bacteria and fungi, respectively). We used the standard antibiotics like vancomycin, gentamycin and amphotericin B as positive controls for Gram-positive, Gram-negative bacteria and fungi, respectively. The concentration of the first well not showing any turbidity was determined as the MIC and the concentration of the first well without any growth on agar medium was reported as the MLC (7).

## 4. Results

The extraction’s yield was higher for *P. spinosa* aqueous extract (1.7% w/w), followed by methanol and ethanol extracts (1.14 and 1% w/w), respectively. The total phenolic and flavonoid contents of ethanol extract (57.7 and 30.6 mg/g) were higher than that of the *P. spinosa* methanol extract (57.8 and 24.4 mg/g). The *P. spinosa* aqueous extract had a lower total phenolic and flavonoid contents compared to the other extracts (33.9 and 3.8 mg/g). In the DPPH assay, the *P. spinosa* ethanol extract IC_50_was lower than the other extracts (82 μg/mL) but was higher than the IC_50_ for BHT (18 μg/mL). The IC_50_ for *P. spinosa* methanol extract was 145 μg/mL, followed by its aqueous extract (550 μg/mL). In the BCB assay, the higher inhibition percent (I%) was for ethanol extract (60%), followed by methanol extract (49.6%) and aqueous extract (36.3%), respectively (Table 1).

The antimicrobial activity of *P. spinosa* extracts showed that the lowest antimicrobial activity was for *P. spinosa* aqueous extracts, followed by methanol extract. The highest antimicrobial activity was for *P. spinosa* ethanol extract. The *P. spinosa* ethanol extract showed a higher activity against *B. cereus* (MIC = MLC = 1.6 mg/mL), *St. mutans* (MIC, MLC = 1.6 and 3.2 mg/mL), *St. sobrinus*, *B. subtilis* (MIC = MLC = 3.2 mg/mL), *St. sanguis* (MIC, MLC = 3.2 and 6.4 mg/mL), *St. salivarius* (MIC = MLC = 6.4 mg/mL) and *S. aureus* (MIC, MLC = 6.4 and 12.8 mg/mL). The *P. spinosa* ethanol extract showed inhibitory effect against *S. epidermidis* (MIC = 3.2 mg/mL). Other microorganisms showed less sensitivity to this extract. The *St. sobrinus* with MIC and MLC 0.8 and 1.6 mg/mL had the most sensitivity to *P. spinosa* aqueous extract, followed by *St. salivarius* (MIC = MLC = 6.4 mg/mL), whereas *St. agalactiae* (MIC, MLC = 3.2 and 6.4 mg/mL) and *B. cereus* (MIC = MLC = 6.4 mg/mL) had more sensitivity to *P. spinosa* methanol extract than the others. Gram-negative bacteria, yeast and fungi were resistant to *P. spinosa* extracts (Table 2).

**Table 1.** Total Phenolic Content, Total Flavonoid Content and Antioxidant Capacity of *Pycnocycla spinosa *Extracts ^a^

Extract	Yield, w/w%	TPC, mg/g	TFC, mg/g	DPPH, IC_50_ μg/mL	ΒCB, I%
**Aqueous**	1.7	33.9	3.8	550	36.3
**Methanol**	1.14	57.8	24.4	145	49.6
**Ethanol**	1.0	57.7	30.6	82	60

^a^ Abbreviations: BCB, β-carotene/linoleic acid bleaching; DPPH, 1,1-diphenyl-2-picrylhydrazyl; TFC, total flavonoid content; TPC, total phenolic content.

**Table 2.** The Antimicrobial Activity of *Pycnocycla spinosa *Extracts ^a, b, c^

	Water		Ethanol		Methanol		Antibiotic	
	MIC	MLC	MIC	MLC	MIC	MLC	MIC	MLC
***S. aureus***	*	*	6.4	12.8	*	*	0.25	0.25
***S. saprophyticus***	*	*	12.8	*	25.6	*	0.25	0.5
***S. epidermidis***	25.6	*	3.2	*	6.4	*	0.5	1
***S. pneumoniae***	*	*	25.6	25.6	25.6	*	0.25	0.5
***S. pyogenes***	*	*	25.6	25.6	25.6	*	0.125	0.25
***S. faecium***	*	*	25.6	*	25.6	*	1	2
***S. faecalis***	*	*	25.6	*	25.6	*	1	2
***S. agalactiae***	*	*	25.6	*	3.2	6.4	0.5	1
***St. mutans***	25.6	25.6	1.6	3.2	25.6	*	0.25	0.25
***St. sobrinus***	0.8	1.6	3.2	3.2	3.2	6.4	0.25	0.5
***St. sanguis***	*	*	3.2	6.4	25.6	25.6	2	4
***St. salivarius***	6.4	6.4	6.4	6.4	25.6	*	0.5	1
***Sh. flexeneri***	25.6	*	25.6	25.6	25.6	*	0.25	0.5
***E. coli***	*	*	25.6	*	25.6	*	0.25	0.5
***S. typhimurium***	*	*	25.6	*	25.6	*	1	2
***K. pneumoniae***	*	*	25.6	25.6	25.6	25.6	0.25	0.25
***Sh. dysenteriae***	25.6	*	25.6	25.6	25.6	*	0.25	0.5
***P. aeruginosa***	*	*	25.6	*	25.6	*	0.25	0.5
***E. aerogenes***	*	*	25.6	*	25.6	*	0.25	0.5
***B. subtilis***	25.6	*	3.2	3.2	25.6	25.6	0.125	0.25
***B. cereus***	25.6	25.6	1.6	1.6	6.4	6.4	0.25	8
***C. albicans***	*	*	25.6	25.6	25.6	25.6	0.125	0.25
***C.glabrata***	25.6	25.6	25.6	25.6	25.6	25.6	0.25	0.5
***A. flavus***	*	*	25.6	25.6	25.6	25.6	0.5	1
***A. parasiticus***	*	*	25.6	25.6	25.6	*	0.5	1
***A. niger***	25.6	*	*	25.6	25.6	25.6	0.5	0.5

^a^ Abbreviations: MIC, minimal inhibitory concentrations; MLC, minimal lethal concentrations.

^b^ Vancomycin, gentamycin and amphotericin B for Gram-positive and Gram negative bacteria and fungi, respectively.

^c^ *, ≥ 52.1 mg/mL.

## 5. Discussion

The results from this study confirmed ethanol as the extraction solvent has an important effect on total phenolic and flavonoid contents of ethanol extract; therefore, it significantly changes the antimicrobial and antioxidant activity of *P. spinosa*. The results from this study showed that *P. spinosa* ethanol extract with higher total phenolic and flavonoid contents showed the lowest IC_50_ value and highest antioxidant and antimicrobial activity. It is reported that phenolic compounds have different biological effects, like anti-inflammatory, antimicrobial, antioxidant effects, cardio protective and vasodilator effects (9-12). There are a positive correlation between total phenolic and flavonoid contents and the antioxidant and antimicrobial activities. The positive correlation between total phenolic and flavonoid contents and the antioxidant effects were observed for *Azilia eryngioides* ethanol extract (13), while this correlation was reverse for *Sambucus nigra* extracts (14). Therefore, the direct correlation between total phenolic content of extract and its antioxidant activity is not an absolute relation. The antioxidant activity of phenolic compounds depend on the molecular weight, the number of aromatic rings and the nature of hydroxyl and conjugate groups (15, 16). Phenolic compounds with higher molecular weight, more hydroxyl and conjugate groups have higher abilities to scavenge free radicals. Therefore, phenolic compounds of *P. spinosa* showed high antioxidant activities.

Higher phenolic and flavonoid contents in *P. spinosa* ethanolic extracts exhibited higher antimicrobial activity, especially against oral Gram-positive bacteria. The Gram-negative bacteria and fungi were resistant to *P. spinosa* extracts while oral *Streptococcus* sp. showed more sensitivity to *P. spinosa* extracts, especially to its ethanol extract. A higher total phenolic content and consequently, a higher antimicrobial activity against fungi was detected for *A. eryngioides* (13), while *P. spinosa* ethanol extract showed less activity against Gram-negative bacteria and fungi. Therefore, different phenolic compounds target different functions of microbial cells. Phenolic and flavonoid compounds inhibit the nucleic acid synthesis (17), cytoplasmic membrane function (18) and energy metabolism (19) of the microbial cell.

The antispasmodic and anti-diarrheal effects of *P. spinosa* ethanol extract were confirmed (2, 20) and these effects are related to isovanillin and isoacetovanillon, through decreasing small intestinal transit of meal (21), induced by 5-HT and EFS (electrical field stimulation) (21). Isoacetovanillon showed a little antibacterial activity against *Escherichia coli* and *Shigella flexneri* (22). This study is the only research about the antibacterial activity of isoacetovanillon, as one of the *P. flexeneri* ethanolic extract components. Therefore, this investigation is the first study about antibacterial and antioxidant activities of *P. spinosa* etxtracts, which can be used as a mouth antiseptic for the treatment of oral streptococcal infections. More clinical studies are essential for evaluating its efficacy in oral infections.
